# Editorial: Reactive oxygen species (ROS) signaling during cytoskeleton dynamics

**DOI:** 10.3389/fcell.2023.1295263

**Published:** 2023-10-04

**Authors:** Lia S. Nakao, Michael F. Olson, José Pablo Vázquez-Medina, Alejandra Valdivia

**Affiliations:** ^1^ Department of Basic Pathology, Federal University of Paraná, Curitiba, PR, Brazil; ^2^ Department of Chemistry and Biology, Toronto Metropolitan University, Toronto, ON, Canada; ^3^ Department of Integrative Biology, University of California, Berkeley, Berkeley, CA, United States; ^4^ Division of Cardiology, Department of Medicine, School of Medicine, Emory University, Atlanta, GA, United States

**Keywords:** ROS, cytoskeleton, actin, NADPH oxidase, oxidation

Reactive Oxygen Species (ROS) such as superoxide radicals (O2•-), hydrogen peroxide (H_2_O_2_), and hydroxyl radicals (•OH) play essential roles in human physiological and pathological processes (Li et al., 2016). Physiological ROS levels regulate fundamental cellular functions, including cell cycle, immune responses, organelle movement, vesicular transport, and migration. Most importantly, excessive ROS levels are often associated with oxidative stress, which may result in RNA/DNA damage, lipid peroxidation, and protein oxidation, leading to tissue damage, aging, and cell death related to various diseases (Brieger et al., 2012). The cytoskeleton is a dynamic and complex network composed of microfilaments, intermediate filaments, and microtubules. The cytoskeleton provides structural support to cells, facilitates cell movement, and is involved in many of the cellular functions mentioned above. ROS plays a pivotal role in modulating cytoskeleton dynamics by directly modifying the cytoskeleton (actin, tubulin, and intermediate filaments) and cytoskeleton-associated proteins (e.g., GTPases, kinases, phosphatases) (Hobbs et al., 2014; Valdivia et al., 2015; Hurst et al., 2022; Zuo et al., 2022).

Major sources of ROS include endogenous enzymes that participate in respiration and metabolic processes in the mitochondria and other organelles, though ROS can also be produced by drugs, radiation, environmental pollutants, and heavy metals (Brieger et al., 2012).

NADPH oxidases (Nox1-5 and Duox1/2) are membrane-associated multi-subunit enzymes that produce ROS (O_2_^•-^ and H_2_O_2_) in various biological systems (Lassegue and Griendling, 2010; Lassegue et al., 2012; Vermot et al., 2021). Nox-derived ROS can regulate important cellular functions supported by the cytoskeleton (Valdivia et al., 2015; Xu et al., 2017). In this context, the current Research Topic features two articles highlighting the relevance of Nox enzymes during actin cytoskeleton dynamics.

First, the article by Valdivia et al., describes a new mechanism by which Nox-1-derived ROS regulate directional cell migration. Here, cells lacking Nox1 show reduced migration and fail to form a single lamellipodium at the leading edge of cells, while the addition of exogenous H_2_O_2_ or Nox1 re-expression rescues this phenotype. Mechanistically, Nox1 deficiency induces PP2A mislocation and inactivation, leading to increased phosphorylation and hyperactivation of the Par3, aPKC, Tiam, and Rac1 polarity complex. Moreover, the authors evaluate the physiological relevance of this signaling pathway using a murine model of femoral artery wire injury to generate neointimal hyperplasia followed by histological analysis. Their results suggest that PP2A and aPKC might contribute to reducing neointima formation observed in Nox1^y/-^ mouse arteries and posit these molecules as possible therapeutic targets.

Second, in their contribution, Richter et al. discover a link between actin dynamics and redox signaling derived from NADPH oxidase 5 (Nox5). Using co-immunoprecipitation and *in situ* proximity ligation assays, the authors first show that Nox5 and actin interact in intact cells. In follow-up experiments, the authors show that actin dynamics modulate Nox5 activity upon calcium stimulation, and that Nox5 increases oxidative modifications in actin, shifting the filamentous to monomeric actin ratio. Finally, the authors show that Nox5 knockdown impairs migration in pancreatic cancer cells, underscoring the importance of Nox5/actin signaling for cell biology.

Additionally, exposure to certain chemical compounds can exacerbate ROS production in cells, leading to pathological migration as may occur in cancer. An example is Bisphenol A (BPA), a widespread chemical used in plastics and resin production, such as those found in food storage containers, beverage bottles, and baby bottles. Small amounts of BPA contaminate foods and drinks, exposing humans daily to its known endocrine-disrupting effects. Although the effect of BPA on cancer cell migration has previously been documented, particularly for ovarian, prostate, breast, lung, and colorectal cancers, the article by Xia et al. shows that the increased colorectal cancer cell migration induced by low nanomolar concentrations of BPA results from exacerbated production of mitochondrial and NOX-derived ROS, activating the HIF1/VEGF pathway. Such increased cell migration may contribute to colorectal cancer progression, particularly since the authors find higher BPA concentrations in the blood of colorectal cancer patients compared to healthy volunteers. The results reported in this article highlight the still unknown yet harmful effects of BPA-derived ROS on cancer cell migration.

Finally, the review article by Tetsuya Ishimoto and Hisashi Mori explores methods for the manipulation of actin polymerization through the generation of ROS using various forms of light and radiation. Actin is a 42 kDa cytoskeletal protein with a highly conserved sequence across organisms. The polymerization and depolymerization of actin regulate essential cellular functions, including motility and morphology. Actin reorganization can be affected via ROS target proteins, including actin and actin-binding proteins. The authors provide an overview of techniques that have been used to control actin polymerization externally, including ultraviolet, visible and near-infrared light, ionizing radiation, and chromophore-assisted light inactivation (CALI). Additionally, they highlight the utility of the fluorescent protein KillerRed and the luminescent protein luciferase to generate ROS on actin fibers, thus promoting polymerization. These methodologies are valuable tools for understanding the relationships between ROS, cytoskeleton regulation, cellular function, movement, and shape. In addition, these approaches also have potential therapeutic applications.

## Conclusion

Together, the articles included in this Research Topic highlight the physiological and pathological relevance of ROS in cytoskeleton remodeling. These articles emphasize that moderate levels of ROS may be involved in normal cellular processes and signaling, such as cell polarity and migration. In contrast, excessive or chronic ROS production may induce detrimental cytoskeletal alterations and contribute to aberrant migration and progression of diseases such as cancer and cardiovascular disease. Additionally, developing new methodologies and tools to study the relationship between ROS and the cytoskeleton can potentially contribute to developing new therapeutic approaches. How ROS modulate the cytoskeleton remains relatively under-studied in many other illnesses, such as neurodegenerative or autoimmune diseases, and we hope that future research will shed more light on the signaling pathways driving these events.
